# Research Progress on Methods for the Deacidification of Small Berry Juice: An Overview

**DOI:** 10.3390/molecules29194779

**Published:** 2024-10-09

**Authors:** Fei Wang, Yao Wang, Xinting Shen, Rui Zhao, Zhebin Li, Jiawu Wu, Huifang Shen, Xinmiao Yao

**Affiliations:** 1 Food Processing Research Institute, Heilongjiang Academy of Agricultural Sciences, Harbin 150086, China; wangf2022822@163.com (F.W.); wang1221yao1221@163.com (Y.W.); 15663585599@163.com (X.S.); lilyamongthorns@163.com (R.Z.); lizhebin2010@163.com (Z.L.); wujiawu1115@163.com (J.W.); 2 Heilongjiang Province Key Laboratory of Food Processing, Harbin 150086, China; 3 Heilongjiang Province Engineering Research Center of Whole Grain Nutritious Food, Harbin 150086, China

**Keywords:** small berry, organic acid, deacidification, key technology

## Abstract

As some of the richest sources of natural antioxidants, small berry fruits have attractive colors and special tastes, with recognized benefits for human health. However, sour tastes in small berry juices result in a poor flavor and low acceptance among consumers, greatly limiting their marketability. Among the most commonly used deacidification methods, chemical deacidification methods can neutralize fruit juice via the addition of a deacidification agent, while physical deacidification methods include freezing deacidification, ion-exchange resin deacidification, electrodialysis deacidification, and chitosan deacidification. All of these methods can markedly improve the pH of fruit juice, but they introduce new substances into the juice that may have an influence on its color, taste, and stability. Biological deacidification can effectively remove malic acid from fruit juice, reducing the content from 15 g/L to 3 g/L; additionally, it maintains the taste and stability of the juice. Therefore, it is widely applied for fruit juice deacidification. On this basis, some compound deacidification technologies have also emerged, but they also present problems such as high costs and complicated working procedures. This review of deacidification methods for small berry juice provides a foundation for the industrial development of such juices.

## 1. Introduction

Small berries represent a very diverse group, including a variety of red, blue, or purple small-sized and highly perishable fruits. Also named soft fruits, this group includes strawberries, currants (black, red, or white) [1], gooseberries, blackberries, raspberries (black or red), blueberries, cranberries, and other berries of minor economic importance (i.e., boysenberries, bilberries, jostaberries, cloudberries, loganberries, and lingonberries) [2]. These fruits are a hot topic in the field of food research at present. They have the characteristics of unique flavors, bright colors, high nutritional value, rich taste, popularity with consumers, and huge market potential [3]. At the same time, berries are rich in phenolic compounds [4], organic acids [5], flavonoids [6], and anthocyanins [7], which have strong antioxidant [8], anti-inflammatory [9], anticancer [10], and hypolipidemic functions [11]. Therefore, small berries are considered potential green and healthy functional foods [12]. However, due to their soft texture, sour taste, and perishable state, berries are often processed into various products, rather than being eaten fresh. The pH of these berries is low, in the range of 2.7–3.6. The sugar content determines the acidity of the flavor, which results from the presence of citric acid and malic acid. In particular, high concentrations of malic acid cause undesirable acidity in the final product.

Juice, as a representative berry product, may meet modern consumers’ demands for fruit juice products. Consumers’ tastes have changed from the simple pursuit of taste in the past to placing equal emphasis on taste and nutritional value. In recent years, a series of foods made from small berries have been successfully launched abroad and have been popular among people who prioritize healthy eating. Because of the high acid content of small berries, many manufacturers produce fruit wines and juices by adding water to balance the taste. The addition of water not only reduces the content of organic acids but also modifies small berries’ characteristic aromas, nutritional components, and health-promoting functions. These influencing factors seriously restrict the development and promotion of small berry products. In this review, we summarize a series of deacidification methods that can be applied to small berry juice, including chemical, physical, biological, and compound deacidification, to provide new ideas for the industrial development of small berry juice.

## 2. Organic Acids in Small Berry Juice

The composition and contents of organic acids influence fruits’ organoleptic properties [13]. A fruit’s organic acid content has a strong relationship with its species, cultivar, cultivation conditions, etc. [14]. The sugar–acid balance and contents are the primary determinants of a fruit’s taste [15]. The acids most commonly found in fruits are citric, malic, and tartaric acids, which affect taste–aroma formation and many physiological processes and are known as “fruit acids” [16,17].

### 2.1. Citric Acid

The most common organic acid in fruits, citric acid has a strong sour taste that can promote saliva secretion and aid digestion; it also has antioxidant and antibacterial effects. In berry fruits, citric acid accounts for 30% to 95% of the total organic acid content. In research by Li et al. [18], strawberry fruits were found to be rich in citric acid, malic acid, succinic acid, and oxalic acid. Li Jiaxiu et al. [19] showed that the organic acid contents of ten strawberry juices mainly comprised citric acid, malic acid, and fumaric acid. Among these, the content of citric acid was the highest, accounting for 62.39~82.73% of the total organic acid content, followed by malic acid, accounting for 16.22~37.51%, and fumaric acid, accounting for only 0.05~0.17%. No tartaric acid was detected. Lerceteau et al. [20] also found that the main organic acids in strawberry fruits were citric acid and malic acid, and the content of citric acid was much higher than that of malic acid, but no fumaric acid was detected in their study. Basson et al. [21] showed that citric acid accounted for two-thirds of the total acid content in strawberry fruits. Cetin et al. [22] determined that the organic acid composition of red raspberry mainly consisted of citric acid, which accounted for 13.1 g/kg (fw), and a small amount of malic acid was also detected. The citric acid content was as high as 10~12 times that of malic acid. Kuang et al. [23] determined the average contents of various organic acids in six kinds of red raspberries. In order from high to low contents, citric acid, lactic acid, DL-malic acid, and oxalic acid were detected. No tartaric acid was detected. The contents of organic acids in the various varieties of red raspberry displayed an obvious difference, but among them, the content of citric acid was the highest, ranging from 1058.41 to 1825.45 mg/100 g. Citric acid, the main organic acid in small berries, has a mild and refreshing sour taste, which endows a pleasant acidity and flavor to fruit juice. However, an excessive content of citric acid causes an unacceptably sour taste. In such cases, it is necessary to adopt certain deacidification methods to improve the acceptance of small berry juice among consumers.

### 2.2. Malic Acid

Malic acid is the second prevailing organic acid in berry fruits [24]. Its acidity is slightly weaker than that of citric acid, but it provides the desired sense of sweet and sour balance. Malic acid can promote metabolism, can help people sweat, and has an anti-fatigue effect. Fu et al. [25] found that the content of DL-malic acid in sea buckthorn juice reached 22.58 mg/mL, about 20 times higher than that of citric acid; this explains why seabuckthorn juice is sour in its taste, with high acid and low sugar contents. In Aronia melanocarpa, the total acid content is 8.20–16.80 g/L [26], among which malic acid is the main organic acid, at 5.60–16.30 g/kg [27]. Malic acid has a pungent and refreshing sour taste, slightly bitter and astringent, with a long-lasting aftertaste. Because of its high content and acidity, malic acid has a strong effect on the flavor of juices and wines. Taking wine as an example, a high content of malic acid may cause wine to ferment in its bottle, leading to a decline in wine quality or even rancidity, which is not conducive to the successful preservation and sale of finished wines [28].

### 2.3. Tartaric Acid

Tartaric acid is abundant in grapes, and its content in wine is relatively high [29,30]. High contents of tartaric acid are also found in fruits of the Ericaceae family, representing up to 17% of the total analyzed organic acids in berry fruits [31]. Tartaric acid is weak in its acidity but has a unique sour taste. It can also promote calcium absorption and help prevent osteoporosis. Bordonaba and Terry [32] found that tartaric acid accounted for the second-highest proportion of acids (after citric acid) in blackcurrant fruit, with a content of 3.42 mg/g. Tartaric acid also has a dominant effect on a wine’s pH. Although the tartaric acid content of small berries can gradually decrease with maturity, some of this acid is still present in mature fruits, resulting in a certain impact on the taste of small berry juice.

All these organic acids not only imbue small berries with a sour and refreshing taste but also have certain health-promoting effects. In addition, small berries may contain other organic acids, such as quinic acid [33], fumaric acid [34], and succinic acid [35]. The total contents of these other acids account for about 3% of the total organic acid content, and the specific components may be different in different varieties and growing environments. The specific types and contents of organic acids found in small berries are shown in Table 1.

## 3. Methods to Reduce the Acidity of Small Berry Juice

### 3.1. Chemical Deacidification

Chemical deacidification usually refers to the addition of basic weak-acid salts to neutralize certain organic acids in fruit juice or fruit wine, thus reducing the product’s acidity. The most common deacidifying agents include calcium carbonate, potassium carbonate, sodium carbonate, and sodium tartrate. Edwin et al. [39] found that the pH of passion fruit juice could be improved by adding calcium carbonate and calcium hydroxide. However, the added calcium carbonate released CO_2_, hindering its thorough mixing with the fruit juice and affecting the juice’s quality. Calcium hydroxide, as an ideal additive, effectively reduced the acidity of raspberry wine via the addition of a combination of CaCO_3_, KHCO_3_, CaCO_3_-KHCO_3_, K_2_C_4_H_4_O_6_, KHCO_3_, and K_2_C_4_H_4_O_6_ using the double salt method, but the limited deacidification effect and ease of precipitation affected the quality of the wine [40]. Although the chemical deacidification method is simple and effective, the chemical reactions involved may affect the taste and color of fruit juice.

### 3.2. Physical Deacidification

#### 3.2.1. Freezing Deacidification

In this method, fruit wine or fruit juice is cooled using freezing equipment; as a result, the tartrates in the wine or juice are crystallized and precipitate. They can then be filtered out, reducing the acidity by removing the equivalent of 0.5~2.0 g/L of tartaric acid [36,41]. The removal of frozen potassium hydrogen tartrate crystals causes a marked reduction in the acid content. This deacidification method is usually carried out in winter and combined with cold filtration, and it is mainly suitable for reducing the acidity of fruit juice with a high tartaric acid content. For example, this method is often applied to wines during cold stability treatment, but it has no significant effect on reducing other organic acids. In this application, no exogenous reagents or strains are introduced, offering high safety, but it has a narrow application scope and is less feasible in actual production.

#### 3.2.2. Ion-Exchange Resin Deacidification

Acidification via ion exchange is a method that reduces acidity by exchanging ions in an ion-exchange resin with acid radical ions in an acid solution [42]. According to the different properties of the exchanged groups, ion-exchange resins can be divided into cation-exchange resins and anion-exchange resins. Yuan et al. [43] used different anion-exchange resins to deacidify sea buckthorn juice and found that different types of ion-exchange resins can adsorb sea buckthorn fruit acids. D941, a weakly basic anion-exchange resin, has a strong adsorption capacity for titratable acid but a weak adsorption capacity for Vc. Its apparent exchange adsorption capacity for titratable acid is 2.70 g/100 mL, with an adsorption equilibrium time of 3 h, a suitable working flow rate of 4 BV/h, a suitable regenerant NaOH concentration of 0.2%, and up to four regeneration cycles. Li et al. [44] found that the removal rate of tartaric acid reached 69.01% when concentrated grape juice was treated using anion-exchange resin 335 at a ratio of 1:6 at 15.57 °C for 4.35 h, and the removal effect of anion-exchange resin 335 was the best among those studied. Ke et al. [45] studied the effects of different deacidification methods on soaked raspberry wine. The results showed that the introduction of D301 macroporous resin at more than 4 g/L reduced the total acid content of the raspberry wine, with the deacidification rate reaching 40%. The resulting wine was clear and mellow. Ion-exchange resin deacidification is widely used in the deacidification of fruit juices and wines because of its advantages of selective separation and easy industrial operation, without any degradation of the fruit juice/wine’s quality due to the introduction of other impurities.

#### 3.2.3. Deacidification via Electrodialysis

Acid reduction by means of electrodialysis refers to the chemical process of moving charged substances through a selective membrane under an electric field. When juice passes through the electric field, H+ ions move through the anode membrane to the cathode, while acid ions move through the cathode membrane to the anode. Both the cathode and anode membranes are unidirectional membranes, so the strongly charged ions can be separated, thus achieving the purpose of acid reduction. Generally speaking, electrodialysis is better at deacidifying citric acid than malic acid [46], and a bipolar membrane is better than a unipolar membrane [47]. ED was studied in the deacidification of juices from several fruits such as cranberries [48], mandarin oranges, passion fruit, tropical fruits (passion fruit, naranjilla, araza, and mulberries), and pineapples [49]. Elodie et al. [50] used electrodialysis with bipolar membranes (EDBM) to deacidify cranberry juice. During 6h of treatment, the pH value of the juice increased from 2.47 to 2.71, with a deacidification rate of 22.84%. Pelletier et al. [51] also used EDBM to deacidify cranberry juice under a pulsed electric field. The treatment increased the pH of the cranberry juice from 2.45 to 2.74 and greatly improved the deacidification rate; additionally, it did not produce any pollutants or waste. This method is expected to be applied as a green and environmentally friendly deacidification method in the future.

#### 3.2.4. Chitosan Deacidification

Chitosan, a natural macromolecular polysaccharide, contains basic polysaccharides with free amino groups from chitin undergoing deacetylation in the presence of concentrated alkali [52]. Therefore, the number of amino groups is related to the degree of deacetylation. The principle of fruit juice deacidification using chitosan is that the amino groups in chitosan react with the carboxyl groups of the organic acids in fruit juice. Given a constant chitosan deacetylation degree, the addition of chitosan has a strong relationship with the degree of deacidification; however, among the organic acids, chitosan mainly adsorbs malic acid and citric acid [53]. This results in a limited deacidification ability and a narrow range of applications. Zhou et al. [54] compared the deacidification effects of chitosan, sodium carbonate, a combination of chitosan and sodium carbonate, calcium carbonate, and the double salt method on blueberry wine. The results showed that the deacidification rate was the highest with 5 g/L calcium carbonate, reaching 26.5%, and the loss of anthocyanins was low. Although the deacidification effect of chitosan is better than that of chemical methods to a certain extent, the color of the final juice product may be affected by its adsorption process. Another limitation is its high cost.

### 3.3. Biological Deacidification

**Figure 1:**
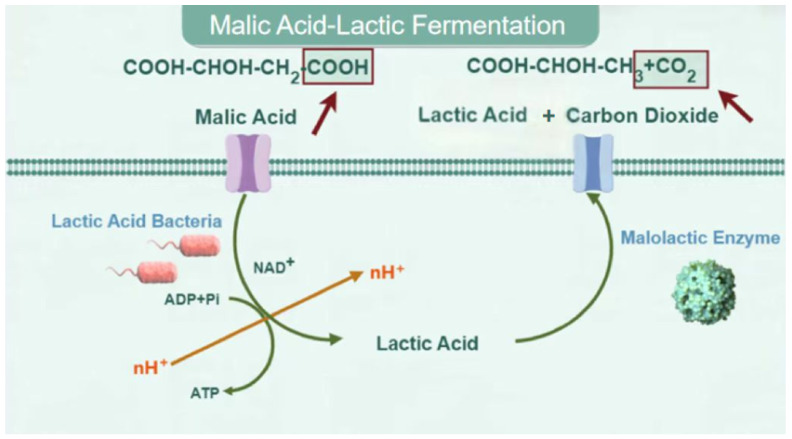
Malic–lactic acid fermentation pathway.

Physical and chemical deacidification methods have no obvious effect on malic acid, but the relatively high contents of malic acid in fruit juices have a strong influence on the juices’ quality and taste [55]. Because of its remarkable deacidification effect, malic–lactic acid fermentation (MLF) has been applied to the development of fruit juice beverages and related products with strong acidity [56,57]. MLF refers to the process in which malic acid is transformed into lactic acid and CO_2_ under MLE in *lactobacillus* (LAB) [58] (Figure 1). Malic acid converts ADP and Pi into ATP under the action of *lactic acid bacteria* and finally forms lactic acid and CO_2_ under the action of enzymes that reduce malic acid. Compared with chemical deacidification methods, MLF can effectively avoid any adverse effects on taste [59]. Katja Tiitinen et al. [60] used *Oenococcus oeni* to reduce the acidity of sea buckthorn juice. First, the raw sea buckthorn juice was diluted 1:1 with water. When the main organic acids in the sea buckthorn juice were fermented for 12 h, more than 50% of the malic acid was converted to lactic acid, resulting in increased astringency. After fermentation for 24 h, the malic acid content decreased from 15 g/L to 3 g/L. However, with continuous extension of the fermentation time, the acidity remained stable and unpleasant flavors were generated. Therefore, this deacidification treatment should be controlled within a short duration. The sugar, Vc, and sea buckthorn oil contents also remained constant during the process of microbial deacidification. Sensory evaluations and chemical composition analyses indicated significant differences between the fermented and unfermented sea buckthorn juice that depended on the variety of the berries, changes in malic acid and lactic acid reactions, sensory changes, and chemical composition [61]. Viljakainen et al. [62] successfully reduced the malic acid and citric acid contents of berry juice by adding *Oenococcus oeni* (ATCC 39401). Their results showed that this organism may deacidify berry juice and wine through the fermentation of malic acid and citric acid. By monitoring the fermentation process, they found that malic acid was quantitatively removed without the loss of any glucose in the berry juice, and its pH changed from 3.5 to 3.7. Lu et al. [63] screened strains *A3* and *B5* with strong deacidification effects on wild raspberries and Lonicera edulis and explored their deacidification effects on berry juices by taking the deacidification rate as an index. The results showed that the deacidification rates of these two strains for malic acid, citric acid, and tartaric acid were 29.76 ± 0.08%, 29.67 ± 0.12%, and 7.42 ± 0.04% and 42.60 ± 0.10%, 18.28 ± 0.15%, and 13.09 ± 0.07%, respectively, within a period of 5 days. *A3* and *B5* were identified as *Hanseniaspora uvarum* and *Zygosaccharomyces bisporus*, respectively, which dominate the field of microbial deacidification.

### 3.4. Compound Deacidification

The existing studies on the deacidification of fruit wine using composite technology include those on the deacidification of Lonicera edulis fruit wine with a combination of calcium carbonate and sodium carbonate [64,65] and blueberry wine with a combination of chitosan and sodium carbonate [54]. There have been studies to screen *Lactobacillus plantarum* and *Saccharomyces cerevisiae* [66], *Lactobacillus plantarum* and *Oenococcus oeni* [67], *Saccharomyces cerevisiae* (SMR-3) and *Schizosaccharomyces* [68], *non-Saccharomyces cerevisiae* (Pichia kudriavzevii NI15) and *Saccharomyces cerevisiae* [69], and *Saccharomyces cerevisiae MH020215* and *Zygo saccharomyces bailiii 749* [70] for wine deacidification. Some biological–physical–chemical methods have also been used to reduce the acidity of fruit wine. However, the current technologies still have problems that need to be solved, such as their high costs and complicated procedures, to allow for improvements and innovation in other fruit juice products. Table 2 summarizes the different deacidification methods applied to fruit juices and wines and compares their deacidification effects. Table 3 summarizes the advantages and disadvantages of the different methods for reducing acids. The differences between them can be understood more intuitively from the table.

## 4. Effects of Deacidification Technology on Fruit Juice Quality

Small berries contain bioactive components such as anthocyanins, polyphenols, and flavonoids, which endow the berries with unique flavors and rich nutritional value. In the process of deacidification, a series of chemical reactions cause changes in the small berries’ bioactive components. The flavor, color, and nutrient content of small berry juice are also changed. In the chemical deacidification of fruit wines, the addition of calcium carbonate may lead to the introduction of excessively many calcium ions into the wine body, causing the wine to taste bitter and astringent. Additionally, calcium carbonate can react with tartaric acid in fruit wine to generate extremely unstable calcium tartrate, thus affecting the stability of the wine after deacidification. This may cause the fruit wine to lose its luster, produce turbidity, and even cause precipitates to form [64]. In contrast, a study of wine deacidification using potassium tartrate indicated that the final quality of the wine was better than that with other deacidifying agents, such as potassium carbonate and calcium carbonate, and the aroma of the fruit wine was well retained [81]. A study by Mc Dougall [82] showed that the total anthocyanin content in fermented sorbus nigricans juice decreased by 99.40% to only 18.40 g mL^−1^, but its aroma components and total phenol content increased. Fermentation with lactic acid bacteria can improve the nutritional properties of small berry juice in terms of polyphenols, flavonoids, and other active ingredients; modify its sensory properties (flavor, color, etc.); and increase its nutritional and health-promoting functions. Furthermore, fermentation and metabolism can generate new substances to increase berry juice’s nutritional and health-promoting functions [83], including its antioxidant capacity. Yang [84] fermented wild cherry juice with lactic acid bacteria and measured the characteristics of the juice before and after fermentation. The results showed that the a*, b*, and L* values of the fermented wild cherry juice increased, indicating that the color of the fermented product was redder, yellower, and brighter than that of untreated juice. However, the overall ∆*E* score showed that this color change during fermentation could not be distinguished with the naked eye. At the same time, the antioxidant capacity and the total phenol and total flavonoid contents of the fermented wild cherry juice were improved, and the number of types of aroma components increased from 24 to 37. The new flavor substances produced were mainly alcohols and esters, including 3-hexyl-1-alcohol, linalool, L-menthol, ethyl hexanoate, and eugenol acetate. Ryu [85] used the *Lactobacillus plantarum GBL17* strain to ferment black raspberry juice with lactic acid. After the fermentation, the contents of total polyphenols and flavonoids in the black raspberry juice had significantly increased, and the DPPH radical scavenging activity of the fermented black raspberry juice (70.92%) was higher than that of the control (62.96%).

## 5. Discussion and Future Perspectives

In summary, the contents and types of organic acids in small berry juice have a strong relationship with the maturity of the raw fruit, and the content of organic acids has a marked influence on the color, taste, flavor, and stability of the resulting juice. The chemical deacidification method is simple and effective, but it may change the color, taste, and stability of the fruit juice due to the incorporation of a large number of metal ions, with effects such as a loss of lightness and turbidity. Physical deacidification methods do not introduce chemical substances and have little influence on the quality of the juice. Resin raw materials have a strong adsorption capacity, easy regeneration, a low cost, and good durability, making them suitable for industrial production. Fruit juice after physical deacidification has a bright color and high clarity, but the process has certain limitations. Most of these methods are high in cost and are mainly suitable for the deacidification of fruit wine; chitosan can also adsorb pigments, resulting in a dullness of color in the final product. Compared with chemical deacidification and physical deacidification, biological deacidification involves natural raw materials and no additives; it is suitable for decomposing malic acid and can also deacidify citric acid. The flavor and nutrients in the fruit juice are kept essentially constant during the deacidification process. At present, compound deacidification has a narrow application range with its high cost and complex technological requirements. This provides considerable space for further explorations of the application of deacidification technologies to small berry juices.

Although biological deacidification is more effective than other deacidification methods and has less of an influence on the quality and nutritional components of small berry juices, it still has some problems. If the method of fermentation followed by deacidification is adopted, although a better deacidification effect and more accurate data can be obtained, there is an influence on the juice quality; if the method of deacidification followed by fermentation is adopted, the fruit juice may become contaminated due to its long-term exposure to the air, which has a certain influence on analyses of the fruit juice’s various physical and chemical indexes. Further research is needed to solve the above problems.

In the future, we should pay more attention to the research and development of deacidification techniques for small berry juices that do not change the flavor, color, or nutritional components of the original juice. At the same time, we must strengthen the research on compound deacidification methods, strive to reduce the cost of deacidification, and simplify deacidification methods in order to deacidify small berry juices in the most efficient and economical way possible. This will provide an excellent foundation for developing the market for small berry juice and wine products.

## Figures and Tables

**Table 1:** Types and contents of organic acids found in different small berries [21,24,36,37,38].

Fruit	Species	Citric Acid (g/kg)	Malic Acid (g/kg)	Tartaric Acid (g/kg)	Fumaric Acid (g/kg)	Oxalic Acid (g/kg)	Total Organic Acids (mmol/kg)
Strawberry	Rosaceae	9.3 ± 0.39	0.98 ± 0.15	-	51.7 ± 6.51	7.9 ± 0.36	57.4 ± 1.9
Blackberry	Rosaceae	5.6 ± 0.42	2.05 ± 0.22	-	34.1 ± 2.54	28.2 ± 2.45	45.1 ± 3.1
Rowanberry	Rosaceae	1.2 ± 0.06	30.28 ± 0.90	0.37 ± 0.03	28.0 ± 1.17	16.1 ± 1.03	235.0 ± 7.2
American cranberry	Ericaceae	14.7 ± 0.86	0.71 ± 0.15	1.968 ± 0.142	35.8 ± 3.24	17.7 ± 0.70	93.9 ± 7.1
Highbush blueberry	Ericaceae	10.3 ± 0.47	0.59 ± 0.06	-	-	27.2 ± 3.84	57.7 ± 8.0
Black mulberry	Moraceae	4.5 ± 0.42	0.74 ± 0.06	-	67.7 ± 2.36	13.6 ± 1.00	29.6 ± 2.6
Goji berry	Solanaceae	2.1 ± 0.28	1.38 ± 0.13	-	11.6 ± 1.45	27.5 ± 3.7	21.5 ± 2.4
Sweet cherry	Rosaceae	0.37 ± 0.17	110.55 ± 261.81	-	112.43 ± 263.51	-	72.17 ± 13.33
Red raspberry	Rosaceae	10.8 ± 0.62	0.94 ± 0.07	0.085 ± 0.013	35.5 ± 2.48	14.2 ± 1.42	5.15 ± 1.27
Bilberry	Ericaceae	5.7 ± 0.32	2.71 ± 0.15	1.852 ± 0.028	-	71.3 ± 4.29	62.5 ± 2.8

**Table 2:** Deacidification methods applied to different fruit juices and wines and the resulting deacidification rates [38,41,46,48,49,56,64,67,68,69,70,71,72,73,74,75,76,77,78,79,80].

Sample		Acid Reduction Method	Acid Reduction Rate
Sea buckthorn juice	Physical deacidification	D941 anion-exchange resin adsorption	Organic acids decreased by 70%
Cranberry juice		Deacidification via electrodialysis	Organic acids decreased by 22.84%
*Schisandra chinensis* juice		Amberlite IRA 67 resin and Lewait MP62-ENG resin	Citric acid decreased by 90%
Dry wild grape wine	Chemical deacidification	Calcium carbonate mixed with potassium bicarbonate	Tartaric acid decreased by 77.8%
Schisandra chinensis juice		CaCO_3_, K_2_CO_3_, KHCO_3_, Na_2_CO_3_	1g Na_2_CO_3_ reduced the total acids by 1.30g/L
Blueberry wine	Biological deacidification	Deacidification with *Saccharomyces cerevisiae*	L-malic acid decreased by 30%
Cherry juice, apple juice, black raspberry juice		*Lactobacillus plantarum* fermentation for deacidification	Tartaric acid decreased by 92%
*Lonicera caerulea* L. juice		Fermentation of *Lactobacillus acidophilus* for deacidification	Organic acids decreased by 86.32%, malic acid decreased by 49.37%, citric acid decreased by 36.05%
Prunus mume		*Lactobacillus* fermentation for deacidification	Titratable acid decreased by 71.4%
Wine	Compound deacidification	*Lactobacillus plantarum* and *Oenococcus oeni*	L-malic acid decreased by 85%
Wild wine		*Saccharomyces cerevisiae (SMR-3)* mixed with *Schizosaccharomyces*	Organic acids decreased by 50%, malic acid decreased by 81.12%
Grape Juice		*non-Saccharomyces cerevisiae (Pichia kudriavzevii NI15)* mixed with *Saccharomyces cerevisiae*	Organic acids decreased by 40%
Wine		*Saccharomyces cerevisiae MH020215* mixed with *Zygo saccharomyces bailiii 749*	Tartaric acid decreased by 43%, organic acids decreased by 12.5%
Kiwifruit wine		Combination of Na_2_CO_3_ and chitosan for deacidification	Organic acids decreased by 44.27%
Cherry wine		Combination of Na_2_CO_3_ and potassium tartrate for deacidification	Organic acids decreased by 38.7%
Indigo fruit wine		Combination of Na_2_CO_3_ and CaCO_3_ for deacidification	Organic acids decreased by 48%
Lemon fruit wine		Weak basic anion-exchange resin D311 combined with *Leuconostoc mesenteroides* fermentation for deacidification	Organic acids decreased by 61%

**Table 3:** The advantages and disadvantages of the leading technologies available.

		Advantages	Disadvantages
Chemical Deacidification	CaCO_3_, K_2_CO_3_, KHCO_3_, Na_2_CO_3_	Fruit juice treated via sodium carbonate deacidification has a strong aroma and suitable taste.	The added chemicals release carbon dioxide, which affects the quality of the juice, easily results in flocculent precipitation, and leads to a poor juice taste and serious aroma loss.
Physical Deacidification	Freezing Deacidification	Does not introduce exogenous substances.	Mostly used to reduce the content of tartaric acid in fruit wine; its application range is narrow.
	Ion-Exchange Resin Deacidification	Selective separation technology does not introduce impurities, ensures the quality of fruit juice/wine, and is convenient for industrial operation.	The cost is high, and it is not suitable for a wide range of applications.
	Deacidification via Electrodialysis	Deacidification is fast, and foreign substances are not added to the fruit juice/wine.	Causes certain loss of flavor substances in fruit juice/wine, the cost is high, and the dialysis membrane is easily fouled.
	Chitosan Deacidification	Chitosan has a large specific surface area, strong adsorption, and a good deacidification effect.	Mainly adsorbs malic acid and citric acid but has poor adsorption effects on other organic acids, so its application has certain limitations.
Biological Deacidification	Malic–Lactic Acid Fermentation (MLF)	Effectively reduces the malic acid content and improves the quality of fruit juice/wine.	Malic–lactic acid fermentation is not suitable for fruit juices/wines with a high sugar content.
Compound Deacidification	Physical–Chemical Deacidification, Chemical–Biological Deacidification, Physical–Biological Deacidification	Reasonable combinations can effectively improve the deacidification rate while improving the flavor and taste of fruit juice/wine.	High costs and complicated procedures render it unsuitable for large-scale industrial application, and many aspects need continuous improvement and innovation.

## Data Availability

Not applicable.
